# Knowledge and attitudes of HIV pre-exposure prophylaxis among nurses in South Africa

**DOI:** 10.4102/phcfm.v15i1.4086

**Published:** 2023-09-19

**Authors:** Veronique C. Bailey, Atholl V. Kleinhans, Mathilda M. Mokgatle

**Affiliations:** 1 Department of Public Health, School of Healthcare Sciences, Sefako Makgatho Health Sciences University, Tshwane, South Africa

**Keywords:** pre-exposure prophylaxis, HIV and/or AIDS, nurses, attitudes, knowledge

## Abstract

**Background:**

Human immunodeficiency virus (HIV) pre-exposure prophylaxis (PrEP) has shown efficacy and effectiveness in populations who practise high-risk sexual activity. Nurses’ knowledge and positive attitudes enhance PrEP implementation.

**Aim:**

This study aimed to investigate the knowledge of and attitudes towards PrEP among nurses in primary health care facilities.

**Setting:**

The study was conducted in 10 health facilities that offer comprehensive services in Tshwane, South Africa.

**Methods:**

A cross-sectional survey assessed the knowledge of and attitudes towards PrEP among 114 nurses. Univariate, bivariate and logistic regressions were performed to estimate odds ratios and to determine whether age, sex and education had an association with the knowledge and attitudes.

**Results:**

Majority of the study sample consisted of female nurses (92.1%), and most respondents (68%) had moderate PrEP knowledge. Logistic regression showed that age and education were not associated with high level of knowledge. Pre-exposure prophylaxis was viewed negatively by 84.5% of the respondents. The odds of positive attitudes towards PrEP were 1.92 times higher among males than females (95% CI 0.54–6.83) and 1.24 times higher among nurses who had bachelor’s degree than diploma holders (95% CI 0.51–3.01).

**Conclusion:**

This study found that there is a need to strengthen the dissemination of information about PrEP, and nurses in South Africa require training to improve their knowledge of and attitudes towards PrEP.

**Contribution:**

The findings of the study add to the current knowledge base regarding PrEP access in the public healthcare system and it highlights gaps in the training of healthcare providers.

## Introduction

The human immunodeficiency virus and/or acquired immune deficiency syndrome (HIV and/or AIDS) pandemic continues to be a major public health concern globally, and particularly in South Africa, where approximately 7 500 000 people are living with HIV.^1^ In 2021, South Africa reported 210 000 new HIV infections and 51 000 AIDS-related deaths.^1^ The search for better and improved HIV prevention methods to control the spread of HIV continues and one such method is HIV pre-exposure prophylaxis (PrEP). Several trials have demonstrated the safety and efficacy of PrEP as a biomedical intervention in the prevention of HIV acquisition.^2,3,4^ In 2015, the World Health Organization (WHO) issued guidelines for the use of oral PrEP. The guidelines recommended that all people who are at high risk of contracting HIV, such as the youth, sex workers and men who have sex with men, should receive oral PrEP, as part of a combination prevention strategy.^5^ In South Africa, PrEP has been included in the National Guidelines on HIV and/or AIDS Treatment and Prevention since 2015.^6^ These guidelines emphasise the use of PrEP as part of a comprehensive prevention strategy, and it is now available in primary care clinics.^7^ PrEPWatch estimates that 569 977 people in South Africa were enrolled as of August 2022.^8^

Pre-exposure prophylaxis is an important public health intervention tool for controlling the HIV epidemic and enabling the ending of HIV transmission.^9^ Nurses are the primary people who will encounter those who could benefit from using PrEP, providing them with information and scheduling regular follow-up and monitoring visits. Nurses must be able to perform a risk assessment, screening and PrEP prescription safely. Nurses must also communicate the importance of adherence, which is critical to PrEP efficacy.^2^

Although there are several studies^10,11,12,13,14^ that assessed healthcare professionals’ knowledge of and views on PrEP, most of these studies were conducted on physicians outside of South Africa. While some nurses are knowledgeable about PrEP, many may lack the necessary information due to a lack of formal training when new programmes are introduced.^10,13,15^ A potential barrier to PrEP uptake is healthcare workers’ limited knowledge, skills and provider attitudes, which has a direct impact on identifying individuals who may benefit from PrEP.^15,16^ As a result, understanding nurses’ knowledge and attitudes about PrEP is critical to the success of the public health rollout of PrEP, as their perspectives can significantly influence public perception and the acceptability of PrEP. This study examines nurses’ knowledge of and attitudes towards PrEP for HIV prevention in Tshwane, South Africa.

## Research methods and design

### Study design

A descriptive, cross-sectional survey design was used to assess nurses’ knowledge of and attitudes towards PrEP for the prevention of HIV infection in Tshwane, South Africa.

### Study setting

The study took place in the primary health care (PHC) facilities located in Tshwane sub-district 1, which include Soshanguve, Mabopane, Ga-Rankuwa, Winterveldt and Akasia. From all the health care facilities in Tshwane North district 1, there are approximately 15 PHC clinics, 3 community healthcare centres (CHCs) and 1 midwife obstetric unit (MOU). Primary health care clinics employ approximately 10 nurses each, CHC employs approximately 50 nurses, and the MOU employs approximately 15 nurses. The services provided at these healthcare facilities include family planning, immunisations, integrated management of childhood illnesses (IMCI), emergency care, HIV counselling and testing, antenatal and/or postnatal care, prevention of the mother-to-child transmission of HIV (PMTCT), chronic care, screening and the treatment of sexually transmitted infections (STIs) and PrEP.^17^ The researcher intended to include all healthcare facilities in the sub-district but instead collected data from only 10 facilities because some facility managers did not grant permission for data collection.

### Study population and sampling

Using convenience sampling, the researchers asked all the nurses who were at the selected facilities at the time of data collection to take part in the study. This method of sampling was a feasible method because the healthcare workers worked in shifts. Eligibility criteria included healthcare workers (doctors and professional nurses) working in a local healthcare setting (clinic) in sub-district 1, Tshwane, regardless of the years of experience, and ability and willingness to provide informed consent for study participation; and excluded healthcare workers not on duty during the date and time of data collection. All healthcare workers from the selected facilities who met the eligibility criteria were invited to participate in the study. Copies of the informational pamphlet were distributed to a total of 127 potential respondents who were available at the date of data collection for their perusal. A total of 114 (*n* = 114) questionnaires were completed in full and considered for data analysis because 13 received questionnaires had to be discarded for various reasons, mainly to being inadequately completed. A total of 114 questionnaires were adequately completed and prepared for analysis. Although the study included both doctors and nurses, only nurses participated during data collection.

### Data collection

An anonymous self-administered paper-based questionnaire was used to collect the data. The questionnaire was adapted from the tool used by the Fenway Institute.^10^ The questionnaire was available in English and contained close-ended questions. Data collection occurred over a period of 3 months from September 2019 to November 2019. Prior to conducting the study, the questionnaire was pretested on seven professional nurses who were conveniently recruited from a local nongovernmental organisation (NGO) to identify any unclear or ambiguous items in the questionnaire and to verify its accuracy. Data collected from the pretesting of the data collection tool were not included in the data analysis of the main study. No problems were identified in the tool and no changes were made.

The questionnaire was divided into three sections and consisted of 32 questions. It included true-or-false questions and Likert scale questions. Section A focused on demographic information which assessed the age, gender, occupation, marital status, highest level of education, race and religion. Section B assessed the PrEP knowledge of nurses, and section C assessed the attitudes towards PrEP held by nurses. Seven questions were asked to determine the level of knowledge. The total number of correct responses to the seven knowledge questions provided an overall assessment of the respondent’s knowledge of PrEP on a seven-point scale. The results were classified as ‘good’ if they scored 7 out of 7, ‘moderate’ if they scored between 4 and 6, and ‘poor’ if the scores were less than 4 on the scale. Twelve statements were used to elicit respondents’ attitudes. When evaluating the general attitude of respondents towards PrEP, the following criteria were applied: respondents were asked to rate their level of agreement with a series of PrEP-related statements as agree, unsure or disagree; a point value ranging from −1 to +1 was assigned to each response option. The possible score range was −12 to +12. A score of 6 or more showed a positive attitude, while a score of less than 6 showed a negative attitude.

To minimise interruptions with the duties of nurses, the researcher arranged with the facility managers to address the potential respondents during their weekly morning meetings. The research assistant administered the surveys to the respondents after the meetings. The respondents required approximately 15 min – 20 min to complete the questionnaire. The respondents placed their confidential questionnaires in a box before exiting the interview venue. A total of 127 questionnaires were distributed and completed; and only 114 were accepted for analysis because 13 of the received questionnaires had to be discarded for a variety of reasons, including being incomplete.

### Data analysis

The data were collected using Epi-Info, which was then exported to STATA for cleaning and analysis. All analyses were performed using STATA version 13 (Stata Corp., College Station, TX, USA). Nonresponses were categorised as missing data, and missing values were omitted from the analysis. The study’s outcome variables were nurses’ knowledge and attitudes regarding PrEP.

For descriptive statistics, continuous variables were summarised using the median with interquartile range. For categorical variables, the frequency and proportion or percentage were used. The Cronbach alpha analysis was applied to verify the reliability and internal consistency of the data collection instrument.^11^ In this study, the knowledge and attitude was treated as a continuous scale, then calculated to full score, and further given cutoff points. The lower scores indicate poor knowledge and negative attitudes, and the Cronbach’s alpha was 0.741 for knowledge and 0.84 for attitudes. Logistic regression was used to compute odds ratios (ORs) and 95% confidence intervals (CI) for the relationship between the level of knowledge of and the attitude towards PrEP and the respondent characteristics. Finally, the Pearson’s chi-square was applied to assess the relationship between knowledge and attitude. All *p*-values less than 0.05 were deemed statistically significant. To assess factors associated with knowledge and attitude, which are the two outcomes of the study, we first collapsed knowledge scores (good, moderate, poor) into two categories, which are good and poor, where moderate score was combined to one. The rationale was to have a binary variable for the knowledge score. It was further categorised attitudes into positive attitudes and negative attitudes.

### Ethical considerations

The researcher provided each potential participant with an information sheet stating the purpose of the study and the procedures involved. The researcher provided clarity on any questions raised by the respondents. A written informed consent form was signed and dated by the researcher and the respondent. Participation was voluntary and respondents were allowed to withdraw at any point during the study. There were no negative consequences for nonparticipation or withdrawal from the study. The researcher did not capture any identifying information, maintaining confidentiality throughout the study. The study was conducted in accordance with the Declaration of Helsinki.

Both the Faculty of Health Care Sciences Research Committee and the University Research Ethics Committee gave ethical approval for this study (Ref: /H/182/2019: PG). The Gauteng Provincial Department of Health and the Tshwane District Department of Health gave permission to conduct the study.

## Results

### Sociodemographic characteristics of respondents

The survey tools were distributed to 127 participants but only 114 completed the questionnaire, resulting in a response rate of 89.8%. The sample included nurses from 10 health facilities providing HIV-PrEP services. The median age of the respondents was 47 years (IQR 38–54). There were more females (*n* = 105; 92.1%) than males (*n* = 8; 7.0%). Only a small number of respondents, that is, 9.7% (*n* = 11) were widowed or divorced, and 3.5% (*n* = 4) lived with their partners. Most respondents (*n* = 73; 64%) held a diploma, while 36% (*n* = 41) held a bachelor’s degree (Table 1).

**TABLE 1:** Sociodemographic characteristics of respondents.

Characteristics	Median		IQR
Age (years)	47		38–57
**Sex**			
Female		105	92.1
Male		8	7.0
Missing value		1	0.9
**Marital status**			
Divorced	11		9.7
Living with partner	4		3.5
Married	50		43.9
Single	36		31.5
Widowed	11		9.7
Missing	2		1.7
**Education**			
Bachelor’s degree		41	36.0
Diploma		73	64.0
**PrEP awareness**			
Yes		108	94.7
No		6	5.3
**Self-rated knowledge of PrEP**			
Good		56	49.1
Fair		40	35.2
Poor		14	12.0
Not sure		4	3.7

PrEP, pre-exposure prophylaxis; IQR, interquartile range.

### Healthcare providers’ knowledge about pre-exposure prophylaxis

**FIGURE 1:**
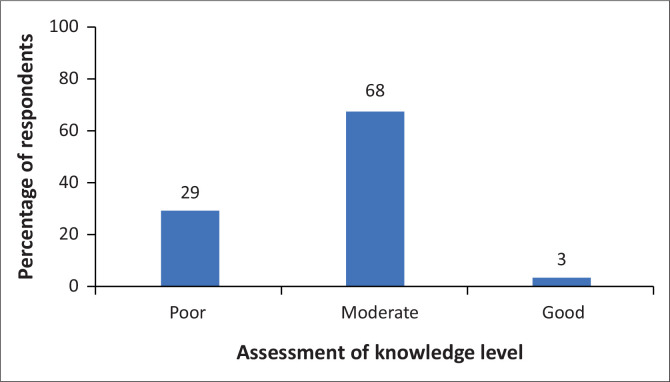
Healthcare providers’ self-rated knowledge about pre-exposure prophylaxis.

Most respondents’ HIV-PrEP knowledge was moderate (68%) and poor (29%), and 3% had good knowledge (Figure 1). However, about half of the respondents (49.1%) assessed their self-rated PrEP knowledge as good and 35.2% as fair (Table 1).

As indicated in Table 1, most respondents reported being aware of PrEP (94.7%). The responses to specific knowledge questions are presented in Table 2. Most respondents knew that PrEP is intended for use as an HIV prevention tool (97.2%, *n* = 105), that it is taken orally once daily (90.8%) and that it has been approved for use in the country (88%). More than half of the respondents (53.7%) correctly identified that PrEP is not intended for both treatment and prevention. Few respondents (34.3%) provided a correct response that PrEP is not intended for use by both HIV-negative and HIV-positive individuals. Most respondents (77.8%) correctly identified that PrEP is not currently available to all potential users in PHC facilities, and that individuals on PrEP do not need to be followed up every month for medication side effects and lab toxicities.

**TABLE 2:** Healthcare providers’ knowledge of pre-exposure prophylaxis.

Knowledge questions	True		False		Correct response
	*N*	%	*N*	%	
1. PrEP refers to antiretroviral (ARV) medication used to prevent HIV?	105	97.2	3	2.8	True
2. PrEP is intended for both treatment and prevention of HIV?	46	42.6	58	53.7	False
3. The combined pill Tenofovir and/or Emtricitabine has been approved for PrEP use in South Africa?	95	88.0	12	11.1	True
4. PrEP is intended for use in both HIV-negative and HIV-positive individuals?	71	65.7	37	34.3	False
5. PrEP is taken orally once daily?	98	90.8	9	8.3	True
6. PrEP is currently available to all potential users in the primary health care facilities?	24	22.2	84	77.8	False
7. Patients on PrEP should be followed up every month for medication side effect and lab toxicities?	26	24.1	80	74.1	False

HIV, human immunodeficiency virus; PrEP, pre-exposure prophylaxis.

Logistic regression indicated that males were 1.53 times more likely than their female counterparts to identify PrEP as an HIV preventive measure (OR = 1.53; 95% CI 0.61–3.85; *p* = 0.363). The odds of recognising PrEP as an HIV prevention measure was the same for the nurses who had a bachelor’s degree and the diploma qualifications (OR = 1.04; 95% CI 0.55–1.96; *p* = 0.902). However, the association between both sex and education and knowledge was not statistically significant. Age was not associated with knowledge of PrEP (Table 3).

**TABLE 3:** Logistic regression comparing pre-exposure prophylaxis knowledge and respondents’ demographic characteristics.

Characteristics	Logistic regression	95% CI	*p* value
Age	1.00	0.98–1.03	0.753
**Sex**			
Female	1	-	-
Male	1.53	0.61–3.85	0.363
**Education**			
Diploma	1	-	-
Bachelor’s degree	1.04	0.55–1.96	0.902

CI, confidence interval.

### Healthcare providers’ attitudes towards pre-exposure prophylaxis

**FIGURE 2:**
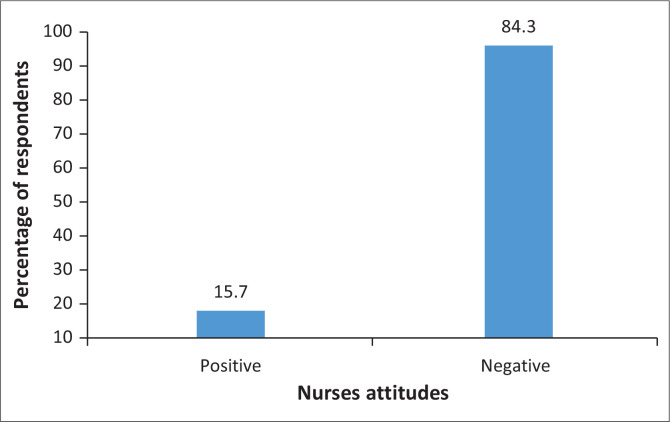
Overall healthcare providers’ attitudes towards pre-exposure prophylaxis.

Overall, HIV-PrEP was viewed negatively by 84.5% of respondents (Figure 2).

Respondents’ attitudes about HIV-PrEP are demonstrated in Table 4. Although the overall attitude was less favourable towards PrEP, the respondents demonstrated positive attitudes regarding providing PrEP education as an essential part of HIV prevention (90.7%), willingness to recommend PrEP to potential users (91.7%), rolling out PrEP in PHC (67.6%) and that using PrEP could empower women who are unable to negotiate condom use (64.8%). Respondents also showed positive attitudes by agreeing that PrEP adherence (34.3% of the respondents) and the dosing regimen (23.3% of the respondents) could be barriers to PrEP use and by disagreeing (66.7% of the respondents) with the statement that it is unethical to give PrEP to healthy people. Negative attitudes were evident in the responses where approximately half of the respondents (53.7%; *n* = 58) responded that the use of PrEP will lead to increased sexual risk-taking behaviour, and 45.5% (*n* = 49) responded that PrEP promotes HIV resistance.

**TABLE 4:** Attitudes towards pre-exposure prophylaxis among healthcare providers.

Attitude statements	Agree		Disagree		Not sure	
	*n*	%	*n*	%	*n*	%
1. PrEP education is an essential part of HIV prevention education.	98	90.7	7	6.5	3	2.8
2. The use of PrEP will lead to increased sexual risk-taking behaviour.	58	53.7	41	38.0	9	8.3
3. PrEP promotes HIV resistance.	49	45.4	33	30.6	26	24.0
4. PrEP may empower women who are unable to negotiate condom use.	70	64.8	32	29.6	6	5.6
5. It is unethical to prescribe PrEP to healthy individuals.	17	15.7	72	66.7	19	17.6
6. Behavioural interventions are more effective than PrEP.	72	66.7	27	25.0	9	8.3
7. PrEP should be rolled out in a primary health care setting.	73	67.6	17	15.7	18	16.7
8. Potential ARV resistance is a potential barrier to PrEP use.	70	64.8	38	35.5	0	-
9. Adherence to PrEP would be a potential barrier to PrEP use.	37	34.3	38	35.2	33	30.5
10. Medication side effects would be a potential barrier to PrEP use.	61	56.5	35	32.4	12	11.1
11. The dosing regimen would be a potential barrier to PrEP use.	25	23.1	60	55.6	22	20.4
12. I would be willing to recommend PrEP to potential users.	99	91.7	6	5.5	3	2.8

HIV, human immunodeficiency virus; PrEP, pre-exposure prophylaxis; ARV, antiretroviral.

Healthcare providers reported negative attitudes regarding PrEP use, holding the view that PrEP could lead to an increase in sexual risk-taking behaviour (53.7%) and HIV resistance (45.4%), and that potential ARV resistance (64.8%) and medication side effects (56.5%) would be potential barriers to PrEP use. In addition, most respondents believed that behavioural interventions were more effective than PrEP (66.7%), which suggests a negative attitude.

The odds of a positive attitude towards PrEP as an HIV preventive measure were 1.92 times higher among males than females (OR = 1.92; 95% CI 0.54–6.83; *p* = 0.311) and 1.24 times higher among respondents with a bachelor’s degree than respondents with a diploma (OR = 1.24; 95% CI 0.51–3.01; *p* = 0.636). However, the association between both sex and education and knowledge was not statistically significant. Age was not associated with attitudes towards PrEP (Table 5).

**TABLE 5:** Logistic regression comparing respondents’ demographic characteristics and attitudes towards pre-exposure prophylaxis.

Characteristics	Odds ratio	95% CI	*p* value
Age	1.00	0.94–1.01	0.130
**Sex**			
Female	1	-	-
Male	1.92	0.54–6.83	0.311
**Education**			
Diploma	1	-	-
Bachelor’s degree	1.24	0.51–3.01	0.636

CI, confidence interval.

## Discussion

This study assessed the knowledge of and attitudes towards PrEP in South Africa’s Tshwane district, among a sample of professional nurses. Self-reported PrEP awareness among professional nurses was high (94.7%) in our study. Similar to a study conducted in Lesotho, which also reported that despite the high levels of awareness of PrEP among healthcare providers, they still lack granular knowledge about the intricacies of PrEP.^17^ The overall level of knowledge about PrEP among respondents was low. However, most respondents reported high levels of perceived knowledge of PrEP (49.1%), with only 12.0% rating their overall knowledge as poor. This is consistent with findings from other settings, including England and Thailand, where healthcare providers reported a higher level of self-perceived knowledge^18,19^ due to prior knowledge about PrEP.^20,21^ It is reported that high levels of perceived knowledge in PrEP are common in healthcare providers involved in HIV education and care. Prior research, however, has shown that prescribing practices do not always neatly correlate with healthcare providers’ knowledge of PrEP.^22,23,24^

In this study, male respondents and those with a bachelor’s degree were more likely to be knowledgeable about PrEP. However, this association was not statistically significant.^25^ It is reported that male participants had higher odds of reporting familiarity with PrEP guidelines than their female counterparts. It could be that the nurses who took part in this study may not have had adequate in-service training, which suggests the need for training to increase knowledge and create positive attitudes.

Overall, the healthcare providers expressed negative attitudes towards PrEP. This contrasts with reports from several other studies, which found that healthcare providers have positive attitudes towards PrEP.^19,20,21,26,27^ Male respondents and healthcare providers with a bachelor’s degree were more likely to have positive attitudes towards PrEP. However, the relationship was not statistically significant.

Similar to our findings, some studies found that PrEP increases risky sexual behaviour leading to STI acquisition,^18^ increased risk of developing HIV drug resistance,^20^ the concern that patients will not adhere to daily PrEP^21^ and the concern that the use of the drug will cause seroconversion.^17^ A study conducted in the United States among HIV providers found that healthcare providers had concerns about PrEP, which made them less likely to write prescriptions for it.^27^ Despite their negative attitudes towards PrEP, 88.9% of the respondents in our study agreed that they would be willing to counsel potential users about PrEP for HIV prevention.

A weak positive relationship between healthcare providers’ knowledge of and attitudes towards PrEP was found. Better knowledge has been linked to greater acceptance and willingness to prescribe PrEP.^12,15,28^

Some limitations must be noted in this study. Firstly, the study employed convenience sampling. This was the most feasible method of sampling because nurses work in shifts. Secondly, the findings of this study have limited generalisability to nurses in South Africa due to the small sample size which lead to a lack of statistical significance. This is due to the fact that only 10 out of 19 managers from the healthcare facilities identified gave permission to access the potential respondents in some of the PHC facilities in the districts. Thirdly, only nurses participated during data collection.

## Conclusions

This study examined the knowledge and attitudes regarding HIV-PrEP among nurses and clearly demonstrated that some of the nurses in this study lacked knowledge of PrEP, which resulted in negative attitudes towards PrEP. The findings and recommendations of this study suggest that training on PrEP needs to be intensified among nurses not only in Tshwane but also throughout the country. The study clearly demonstrates that more PrEP information needs to be shared with nurses to specifically address some of the common misconceptions identified in this study.
